# How context influences the interpretation of facial expressions: a source localization high-density EEG study on the “Kuleshov effect”

**DOI:** 10.1038/s41598-018-37786-y

**Published:** 2019-02-14

**Authors:** Marta Calbi, Francesca Siri, Katrin Heimann, Daniel Barratt, Vittorio Gallese, Anna Kolesnikov, Maria Alessandra Umiltà

**Affiliations:** 1 Department of Medicine and Surgery, Unit of Neuroscience, University of Parma, Parma, Italy; 2 Interacting Minds Center, University of Aarhus, Aarhus, Denmark; 3 Department of Management, Society and Communication, Copenhagen Business School, Copenhagen, Denmark; 4 Institute of Philosophy, School of Advanced Study, University of London, London, UK; 5 Department of Humanities, Social Sciences and Cultural Industries, University of Parma, Parma, Italy; 6 Department of Food and Drug Sciences, University of Parma, Parma, Italy

## Abstract

Few studies have explored the specificities of contextual modulations of the processing of facial expressions at a neuronal level. This study fills this gap by employing an original paradigm, based on a version of the filmic “Kuleshov effect”. High-density EEG was recorded while participants watched film sequences consisting of three shots: the close-up of a target person’s neutral face (Face_1), the scene that the target person was looking at (happy, fearful, or neutral), and another close-up of the same target person’s neutral face (Face_2). The participants’ task was to rate both valence and arousal, and subsequently to categorize the target person’s emotional state. The results indicate that despite a significant behavioural ‘context’ effect, the electrophysiological indexes still indicate that the face is evaluated as neutral. Specifically, Face_2 elicited a high amplitude N170 when preceded by neutral contexts, and a high amplitude Late Positive Potential (LPP) when preceded by emotional contexts, thus showing sensitivity to the evaluative congruence (N170) and incongruence (LPP) between context and Face_2. The LPP activity was mainly underpinned by brain regions involved in facial expressions and emotion recognition processing. Our results shed new light on temporal and neural correlates of context-sensitivity in the interpretation of facial expressions.

## Introduction

Faces and facial expressions are of utmost importance during social interactions as they provide key signals for understanding the emotional and mental states of others^1–4^. Indeed, in the past decades a growing body of literature has appeared in the field of Affective Neuroscience addressing the neural mechanisms underpinning face processing.

Research into the neural correlates of face processing has revealed a distributed network of cerebral areas encompassing occipito-temporal visual regions such as the middle fusiform gyrus (i.e., fusiform face area (FFA)), the lateral inferior occipital gyrus (i.e., the occipital face area (OFA)), and the superior temporal sulcus (STS). These areas process both the invariant (FFA and OFA) and variant (STS) aspects of facial expressions^4–7^. Other brain regions engaged during the processing of facial expressions include the amygdala, insula, anterior and posterior cingulate cortices, somatosensory areas, and inferior frontal gyrus (IFG)^4,6–10^.

Numerous event-related potential (ERP) studies have shed light on the time course of face processing, showing that the structural encoding is indicated by the N170, a negative component peaking between 140 and 230 ms after stimulus presentation at occipito-temporal sites^11–13^. Whether the N170 can be modulated by emotional facial expressions remains unclear, given previous conflicting results^14–17^. In contrast, the elaboration of semantic and affective information embedded in faces seems to develop at later stages, as indexed by the Early Posterior Negativity, the P300, the N400 and the Late Positive Potential (LPP). Recorded at centro-parietal sites from about 300 to 800 ms after stimulus presentation, the LPP amplitude is usually higher for emotional faces (both pleasant and unpleasant) than for neutral ones. More specifically, as the LPP is higher for both unpleasant and pleasant stimuli with high emotional arousal, this effect is associated with emotional intensity as well as with the increased attentional engagement elicited by such stimuli^18–21^.

It is noteworthy, however, that, although previous studies generally used isolated facial expressions as stimuli, in our daily interactions faces are perceived within particular contexts consisting of emotional body language, surrounding environments, and our intentions and expectations^22–26^. Former ERP studies aiming to investigate the time course of the integration process between contextual cues and facial expressions mainly employed affective priming paradigms^27^ or congruence-incongruence paradigms consisting of: face-body compound stimuli or faces preceded by emotional body postures^28,29^, faces presented in/on an emotive background^30–32^, and faces preceded by emotive sentences^33^. These studies generally showed that contextual cues modulate the N170, with higher amplitudes for faces congruent with the emotional context^27,30,31^. This effect is even more pronounced in later stages of face processing, as indicated by the LPP. Affective priming studies have demonstrated that the LPP is affected by congruence between the prime (e.g., pictures or sentences) and the target (e.g., facial expressions), with higher amplitudes in response to incongruent target stimuli^27,33–36^. For instance, a study by Diéguez-Risco *et al*.^33^ presented sentences (describing happiness- or anger-inducing situations) before the appearance of a congruent or incongruent emotional face, and showed that the LPP can be influenced by preceding contextual information, contrary to early stages of face processing which were unaffected by contextual modulation.

With the purpose of investigating the influence of situational context on the interpretation of facial expressions, Calbi *et al*.^37^ developed an original behavioural paradigm based on a version of the filmic “Kuleshov effect”. Lev Kuleshov (1899–1970) was a Soviet filmmaker who designed an experiment in which he alternated the same close-up of a Russian actor (who had a neutral face) (defined as “Glance shot” in accordance with the *point-of-view editing* (POV) perspective adopted by Barratt *et al*.^38^), with three different emotional contexts (defined as “Object shot” in accordance with POV)^37–39^. Anecdotal reports claim that the viewers of the three film sequences perceived the actor’s face as expressing an emotion congruent with the preceding context^37,39,40^. However, previous experiments used mainly static images as stimuli or adopted experimental designs based on different (non-POV) versions of the Kuleshov experiment^37,38,41,42^.

In Calbi *et al*.^37^, participants were shown film sequences created by editing together three different shots: the zoom-in of the close-up of a target person’s neutral face (first Glance shot), followed by a view of the scene that the target person was looking at (Object shot: happy, fearful, or neutral), followed by another zoom-in of the close-up of the target person’s neutral face (second Glance shot)^37^. With the purpose of investigating the sensitiveness of emotions to different contexts under more ecological conditions, the sequences were constructed with the aim of creating dynamic and spatiotemporal continuity between the glance and object shots^37,38^. Moreover, the use of a triadic structure can be considered as another element of novelty. Unlike in previous experimental paradigms, the first face is shown which may give the impression that the actor is looking at the object shot, and thus strengthen the influence of context. While this interpretation should be further investigated, it is based on Carroll and Persson’s theoretical analysis of the POV structure “*as an instance of deictic gaze*”^38^ (*it duplicates the natural human and primate tendency to follow (from an egocentric position) the gaze of an intentional agent to an object in the adjacent environment*)^38^ (p. 3) and on the necessary conditions to boost the likelihood that the observer makes such an inference^37,38,43,44^.

Participants were asked to rate the target person’s emotion in terms of valence, arousal, and category. Results showed a significant effect of context on both valence and arousal in the fear condition only. Moreover, participants categorized the target person’s emotion choosing emotional categories pertinent with the preceding context^37^.

In the present study, a novel electroencephalographic (EEG) paradigm was used in order to investigate for the first time the neural correlates of the aforementioned contextual effect and its time course. During a high-density EEG recording session, participants were shown 288 film sequences analogous to those in Calbi *et al*.^37^. Participants were instructed to rate both valence and arousal of the target person’s emotion in Face_2. During a subsequent behavioural session, the participants’ task was to explicitly identify the target person’s emotional state based on seven pre-selected categories.

Through the analysis of the EEG activity evoked by Face_2, we assessed the neural networks underpinning this “Kuleshov effect” and its time course. We expected to find a significant modulation of both the N170 and the LPP, due to the influence of the emotional context on face processing. Specifically, considering both the sequentiality of the three shots and the evaluative task requested of the participants, we had two main hypotheses: (1) if the context modulates the perception of the subsequent Face_2, leading participants to perceive it as emotional, then the N170 and the LPP should show higher amplitudes for neutral faces preceded by emotional contexts (presumably higher for the more salient fear condition in respect to the happiness condition); and (2) if the effect of context is not as strong, possibly due to participants’ expectations based on the context itself, then the two ERP components will be modulated by the affective or evaluative congruence between the Object shot and Face_2; namely, the N170 should show higher amplitude for neutral faces preceded by neutral contexts (congruence) and the LPP should show higher amplitude for neutral faces preceded by emotional contexts (incongruence). Hence, according to our second hypothesis, both the N170 and the LPP should not show any difference between the two emotional conditions.

## Materials and Methods

### Participants

Twenty-four volunteers, without formal education in cinema, took part in the EEG and behavioural experiments: 11 female, 13 male, mean age 24.50 years (standard deviation, *SD* =2.21), mean years of schooling 14.92 (*SD* =1.95). All participants had normal or corrected-to-normal visual acuity, no history of neurological or psychiatric impairments and were right-handed, as ascertained by the Edinburgh Handedness Inventory^45^. Five participants were discarded from the EEG analysis due to excessive artefacts. The final sample consisted of 19 participants: eight female, 11 male, mean age 24.11 years (*SD* =2.26), mean years of schooling 14.95 (*SD* =2.07). All participants provided a written informed consent to participate in the study, which was approved by the Institutional Review Board of the University of Parma and was conducted in accordance with the Declaration of Helsinki (2013).

### Stimuli and Procedure

#### Stimuli

The stimuli were composed of film sequences created by editing together three different shots: the close-up of a target person’s neutral face (Glance shot, Face_1), followed by a view of the scene that the target person was looking at (Object shot), followed by a second close-up of the same target person’s neutral face (Glance shot, Face_2)^37,38^.

In creating the sequence, we used neutral faces (12 female, 12 male) selected and digitally manipulated by Barratt and colleagues^37,38^ (taken from the Karolinska Directed Emotional Faces picture set - KDEF^46^), following the procedure described by Calbi *et al*.^37^, thus obtaining 24 faces looking to the right and 24 faces looking to the left (each image lasting 1500 ms).

For the Object shots, we used 48 dynamic scenes (grey-scaled and with sound removed), each with a length of 3000 ms, corresponding to three emotional conditions: Neutral (N = 16), Fear (N = 16), and Happiness (N = 16)^37^.

The three different shots were then joined together into a 6000 millisecond-long sequence consisting of: the first Glance shot (Face_1) presented for 1500 ms, followed by the Object shot presented for a longer duration (3000 ms), followed by the second Glance shot (Face_2) presented for 1500 ms. The sequences were presented in Audio Video Interleave (AVI) format and the resolution of the image was 640 × 480 pixels^37^.

The stimuli included 288 sequences in total, comprising 96 film sequences per emotional condition (Neutral, Fear, or Happiness, in accordance with the emotion evoked by the Object shot). Each facial identity was repeated the same number of times (resulting in 12 repetitions for each identity), both the gender and the orientation of the faces was balanced^37^, and each facial identity could be paired with each context only once.

#### Procedure

A day before the experiment, the participants were asked to fill in several questionnaires via Google Forms. For details regarding questionnaires and participants’ scores, see Supplementary Information.

The entire experiment consisted of two parts: (1) the EEG recording session; and (2) the categorization task.

#### EEG recording session

Firstly, participants were shown 288 film sequences and were instructed to rate, as accurately and as quickly as possible, both the valence and arousal of the target person’s emotion by means of a 9-point scale ranging from −4 (“negative”) to +4 (“positive”) for valence, and from 1 (“calm”) to 9 (“excited”) for arousal^37,38^.

**Figure 1:**
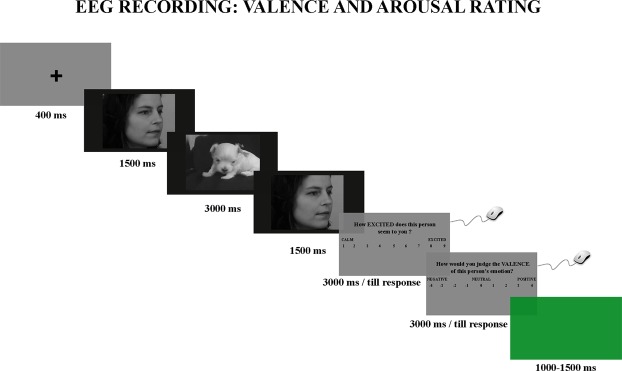
Experimental paradigm. The neutral faces were taken from the Karolinska Directed Emotional Faces picture set – KDEF. The depicted face is AF06NEHR. The frame of the puppy was taken from the video “Cute Puppies!” of “Outstanding Videos” Youtube channel (https://www.youtube.com/watch?v=3RkKvf12Bw0).

More specifically, to rate valence, participants were asked, “How would you judge the *valence* of this person’s emotion?”, while to rate the arousal they were asked, “How *excited* does this person seem to you?” (translated from Italian; Fig. 1). Participants were instructed to move and click the left mouse button with their right hand, selecting the answer in accordance with their first impression. The two questions were presented in random sequence for a maximum of 3000 ms or until the participant responded.

Each trial started with a black fixation cross on a grey background (400 ms), followed by the film sequence presented for 6000 ms on a black background. At the end of the film sequence, the participants’ task was to rate both the valence and the arousal of the target person’s emotion (see above). A green background was used as the inter-trial interval (ITI) with a duration of either 1000 or 1500 ms (Fig. 1).

The experimental session was divided into six experimental blocks (randomly presented), each composed of 48 trials, comprising 16 trials for each condition (both gender and orientation of the faces were balanced). Each Object shot was presented once per block, hence six times in total.

Each experimental block lasted approximately 12 minutes, consisting of a randomised trial order and a rest period of five minutes between each block. Stimuli were shown at the centre of a 19-inch computer screen positioned at a distance of 57 cm from participants.

#### Categorization task

The second experimental part immediately followed the EEG recording session. Participants saw the same film sequences one more time, divided in six experimental blocks (randomly presented), each consisting of 48 trials (see above). In each experimental block, trials were randomly presented and were structured analogously to the EEG experiment (see above). This time, the participants’ task was to explicitly categorize the target person’s emotion, choosing among seven categories (happiness, sadness, fear, anger, disgust, surprise, “other option”)^37^. They articulated their choice by using the keyboard positioned in front of them. No time limit was given. When they chose the “other” option, they had the possibility to write down what was, in their opinion, the perceived emotion^37^.

Stimuli were shown at the centre of a 19-inch computer screen positioned at a distance of 57 cm from the participant.

The experimental session was preceded by a training session which consisted of eight trials (randomly presented). These trials were comprised of film sequences (two neutrals, one happiness, and one fear, each presented twice) composed of object shots excluded at the end of the validation process^37^, as well as four other faces (two female) taken from the KDEF (half of them looking to the left and the other half to the right).

For both experiments, stimulus delivery and response recording were controlled using E-Prime 2.0 software.

At the end of the procedure, participants were asked to answer six open-ended questions via Google Forms to assess their previous experience and their familiarity with the stimuli: (1) Have you ever seen these videos before?, (2) What do you think the experiment was about?, (3) Was there anything confusing in the experiment?, (4) What was your impression of the different faces?, (5) Do you have any other comments?, and (6) Have you heard of the Soviet filmmaker Lev Kuleshov and/or the “Kuleshov effect”?^37,38^.

### EEG Recording and Pre-processing

Continuous EEG was recorded using a 128-channel Geodesic high-density EEG System (Electrical Geodesics Inc., Eugene, OR, USA) through a pre-cabled HydroCel Geodesic Sensor Net (HCGSN-128) at a sampling rate of 500 Hz with the vertex as online reference; sensor-skin impedances were maintained below 50 kΩ for each sensor.

A band-pass filter (1–30 Hz; Notch 50 Hz) was applied on continuous EEG recordings, which were then segmented into epochs lasting 6100 ms (from 100 ms before to 6000 ms after the onset of Face_1) by means of NetStation software (Electrical Geodesics, Inc., Eugene, OR, USA). In order to detect and remove components whose topography, power spectrum and time-course were related to ocular, cardiac, and muscular artefacts, the epoch-file of each participant was imported into EEGLAB toolbox and analysed by means of Independent Component Analysis (ICA)^47^. The resulting IC weights were then applied to raw data filtered with a band-pass filter of 0.5–30 Hz (Notch 50 Hz), in accordance with the observation that such a high-pass filter does not remove or drastically alter slow wave-activity, such as the LPP investigated in the present study^21^. A mean number of 10.8 (*SD* = 0.92) components were removed.

**Figure 2:**
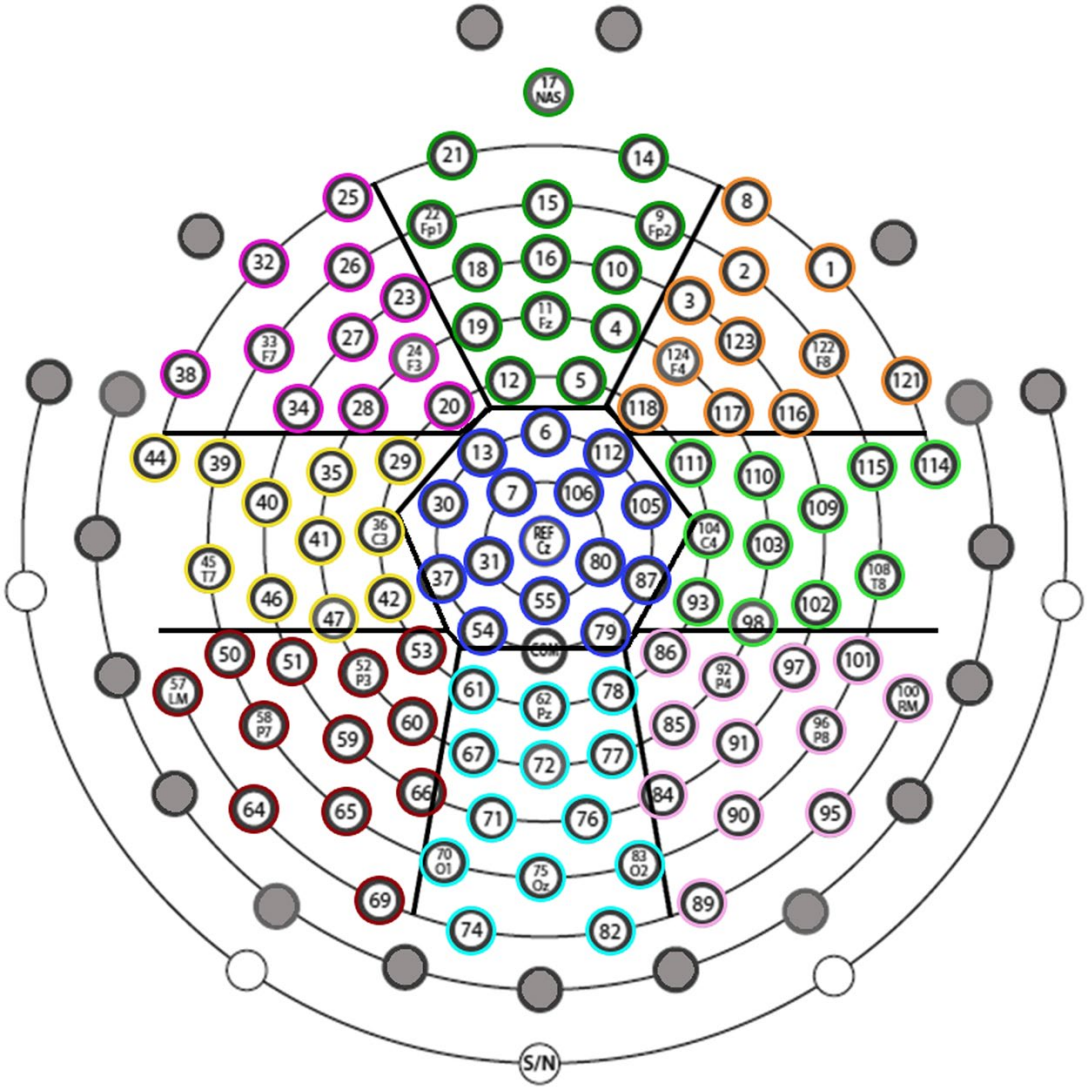
Hydrocel Geodesic Sensor Net – 128 channel map. Grey indicates the outermost belt of electrodes of the sensor net that was excluded from analyses. Other colours indicate the nine different clusters of electrodes considered for global ERP waveform analysis.

The commonly used procedure of channel reduction from 128 to 110 electrodes was employed. The outermost belt of electrodes of the sensor-net (19 peripheral channels: E43, E48, E49, E56, E63, E68, E73, E81, E88, E94, E99, E107, E113, E119, E120, E125, E126, E127, E128) was discarded due to their tendency to show residual muscular artefacts (Fig. 2)^29,48–50^. Bad channels were interpolated using a spherical interpolation method implemented in EEGLAB. The resulting epoch-files were further visually inspected to exclude remaining bad trials (the amount of removed trials was less than 22%) and re-referenced against the average signal of all electrodes.

The means of accepted trials for each experimental condition were: 81.32 (*SD* = 3.86) for the Happiness condition, 81.47 (*SD* = 2.39) for the Fear Condition, and 80.47 (*SD* = 5.28) for the Neutral condition. A repeated-measures ANOVA analysis was performed in order to exclude differences in the number of accepted trials among conditions, which did not result in significance (F_(2,36)_ = 0.41, *p* = 0.67).

Pre-processed data for each participant was subsequently imported and analysed in Cartool software (version 3.55; http://brainmapping.unige.ch). To evaluate Face_1- and Face_2 - elicited ERPs, epochs from the onset of Face_1 and Face_2 up to 1200 ms were averaged across trials, separately for each participant and condition; these single-participant averages were then used to compute three group-averaged ERPs, one for each experimental condition (Fear, Happiness, and Neutral)^29,50^.

#### EEG analyses

EEG data were subjected to two analytic procedures: a global ERP waveform analysis and a global scalp electric-field analysis.

The global ERP waveform analysis was executed as a prior step in establishing the time course of ERP response modulations for both Face_1 and Face_2^51,52^. It was carried out by means of point-wise paired *t*-tests of single-subject ERP average amplitudes of the two compared conditions at each electrode and time-point. Comparisons were performed between: (1) Fear vs. Neutral, (2) Happiness vs. Neutral, and (3) Fear vs. Happiness. The statistical significance level was set at *p* < 0.01 and a 10 contiguous data point temporal criterion (20 ms at our 500 Hz sampling rate) for the persistence of significant effects was applied^53^. Only differences covering at least five adjacent electrodes within nine clusters (see Fig. 2) reaching the statistical significance level were retained^29,50^. In order to control for multiple comparisons, a point-wise paired randomization test was performed (*p* < 0.01; 10 contiguous data point of temporal criterion)^48^. Additionally, in order to compare the three experimental conditions together, we performed two repeated-measures ANOVAs on N170 and LPP mean amplitude values, respectively. For more details, see the Supplementary Information file.

Two statistical analyses were performed on the global electric field for Face_2, enabling a neurophysiological interpretation of significant ERP modulations: a) evaluation of modulations in electric field strength, as measured by the instantaneous Global Field Power (GFP), which reflects quantitative changes in terms of amplitude modulation of statistically indistinguishable generators between experimental conditions; and b) assessment of modulations in electric field topography, measuring the global spatial dissimilarity index (DISS), which reflects qualitative changes in the underlying active brain source configuration^29,50,51,54^. As above, comparisons were performed between: (1) Fear vs. Neutral, (2) Happiness vs. Neutral and (3) Fear vs. Happiness.

Modulations in GFP and DISS between experimental conditions were assessed by non-parametric statistical analyses based on point-wise paired randomization tests^55^. In the present study, the significance level was set at *p* < 0.01, with an additional temporal stability acceptance criterion of 20 ms of consecutive significant difference^29,50,53^.

Variations in electric field strength were measured by means of the statistical comparison of the GFP (the spatial standard deviation of all electrode potentials at a given time-point) between compared conditions for each participant^50,51,54^.

Changes in electric field topography were assessed by DISS, which is a strength-independent index of configuration differences between two electric fields. It is calculated as the square root of the mean of the squared differences between the instantaneous voltage potentials (measured versus the average reference) across the electrode montage (each of which is first scaled to unitary strength by dividing it by the instantaneous GFP). Point-wise paired randomizations performed on the DISS data is also known as “topographic analysis of variance” (TANOVA)^50,51^.

Intracranial sources were estimated in TANOVA time periods corresponding to the latency range of the LPP. We applied a distributed linear inverse solution based on a Local Auto-Regressive Average (LAURA) regularization approach^29,50,56^. The LAURA model recreates the brain electric activity in each point of a 3D grid of solution points, choosing the source configuration that better mimics the biophysical behaviour of electric fields without a priori assumption on the number of dipoles in the brain. The solution space was calculated on a locally spherical head model with anatomical constraints (L-SMAC)^57^ and was comprised of 5018 solution points (voxels) homogeneously distributed within the brain structures of the Montreal Neurological Institute (MNI152) average brain. All solution points were labelled with their Talairach and Tournoux coordinates^58^ as well as their anatomical labels^50^. Intracranial source estimations for each participant and condition over the LPP time window defined by the TANOVA were statistically compared by means of a “voxel-wise parametric mapping analysis”^29,50,59^. To achieve this, individual ERP data were averaged over the period of significant LPP topographic modulation, in order to generate a single data point for each participant and condition^29,50^. LAURA source estimations for each solution point, normalized by root mean square, were then contrasted by means of paired *t-*tests. Solution points with *p* values < 0.01 (*t*_(18)_ > 2.88/< −2.88) were considered significant; additionally, a cluster threshold of at least 10 contiguous activated solution points was applied^29,50^. Source and statistical analyses were performed using Cartool software^29,50,52^.

#### Behavioural analysis

The analysis was performed on data from 18 participants, because of the exclusion of five participants from the EEG analysis and of one participant for technical problems during data acquisition.

In accordance with previous studies by Barratt and colleagues^38^ and Calbi and colleagues^37^, we rescaled valence and arousal scores for each participant so that a value of zero corresponded to the mean rating across all three conditions respectively. This was done in order to evaluate whether, for each participant, a condition mean was higher (positive value) or lower (negative value) than the overall mean in terms of valence and arousal.

In order to investigate the modulation of rating by context condition, we performed a linear mixed effects analysis. We entered the rating score as a dependent variable, and Context (3 levels: Neutral, Fearful, and Happy) and Measure (2 levels: Arousal and Valence) as independent fixed variables. We entered intercept and Context by Measure slope as random effects across participants.

Tukey’s test was used for post-hoc comparisons among means. Visual inspection of residual plots did not reveal any obvious deviations from homoscedasticity or normality. For all analyses, we used R (R Core Team, 2012) and lme^60^.

Regarding the categorization task, we computed the percentage of answers given by participants to each emotion category (Happiness, Sadness, Fear, Anger, Disgust, Surprise, Other emotion) for each emotional condition (for details about statistical analysis see the Supplementary Information file).

## Results

**Figure 3:**
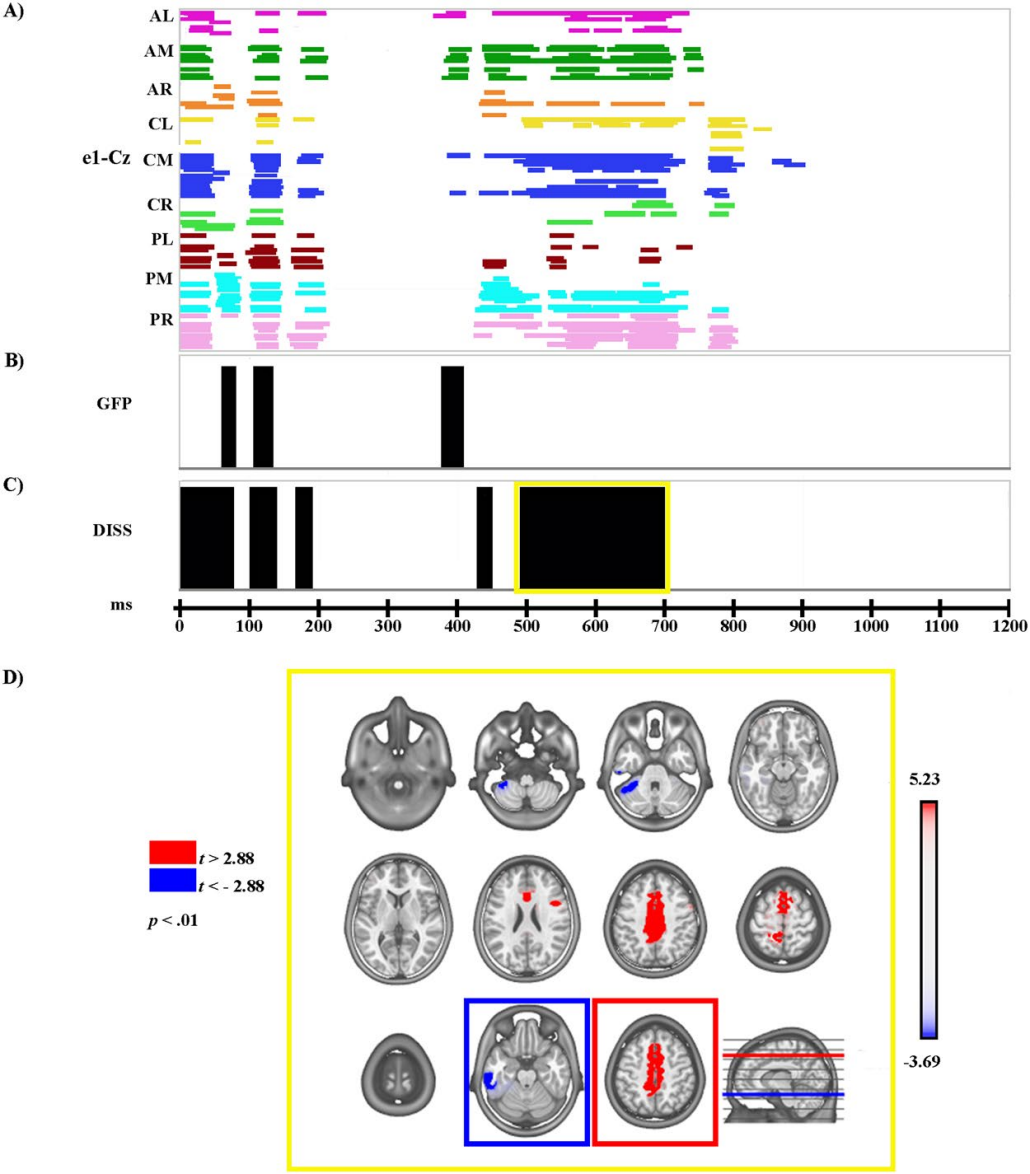
Electrophysiological results and statistical comparison of LAURA source estimation between Fear and Neutral over significant TANOVA LPP time interval. (**A**) Statistical analysis of global ERP amplitude. Periods of significant differences of ERP amplitude (*p* < 0.01; duration >20 ms) at each electrode and time-point between conditions are displayed as coloured horizontal lines. Each horizontal line represents one scalp electrode. Different colours indicate different clusters of electrodes (as shown in Fig. 2); AL: anterior left; AM: anterior midline; AR: anterior right. CL: central left; CM: central midline; CR: central right. PL: posterior left; PM: posterior midline; PR: posterior right. (**B**) Global scalp electric field analysis: statistical analysis of global electric field strength. Black areas indicate time intervals of significant differences (*p* < 0.01; duration >20 ms) of Global Field Power (GFP) between conditions. (**C**) Global scalp electric field analysis: statistical analysis of global electric field topography (topographic analysis of variance, TANOVA). Black areas indicate time intervals of significant differences (*p* < 0.01; duration >20 ms) of global spatial dissimilarity index (DISS) between conditions. (**D**) Significant TANOVA time interval (494–702 ms after Face_2 onset). All significant voxels are coloured (t_(18)_ >2.88/< −2.88, *p* < 0.01): positive *t* values (red) indicate higher current source densities in Fear than in Neutral; negative *t* values (blue) indicate higher current source densities in Neutral than in Fear. LAURA solutions are rendered on MNI152 template brain (left hemisphere on the left side).

**Figure 4:**
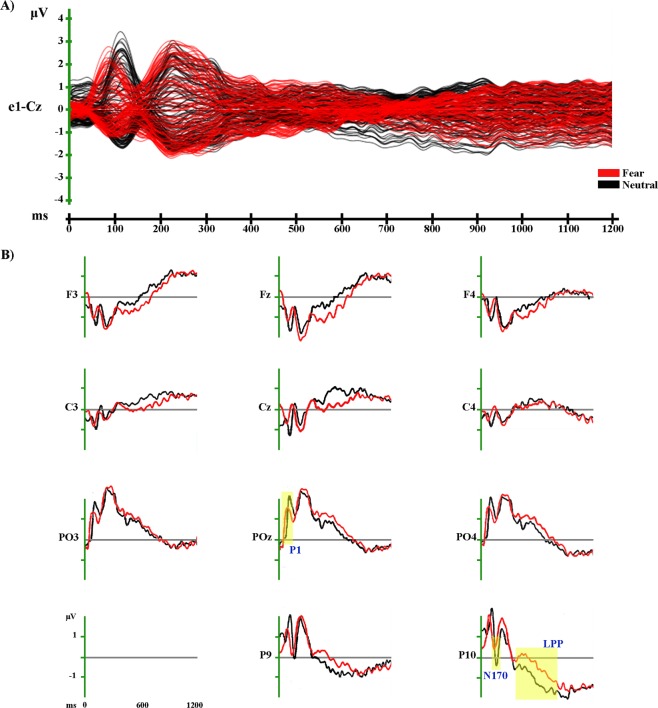
Grand-averaged ERP waveforms of Fear and Neutral conditions. (**A**) Group-averaged (n = 19) event related potential (ERP) waveforms of the two experimental conditions (Fear and Neutral), superimposed across the 110 recording channels (e1-Cz). (**B**) Group-averaged (n = 19) Face_2-locked ERP waveforms recorded at left, midline and right scalp sites (frontal: F3, Fz, F4; central: C3, Cz, C4; parieto-occipital: PO3, POz, PO4) and at left and right occipito-temporal scalp sites (P9, P10), plotted as voltage in µv and as function of time in ms (Face_2: 0 ms). Black: Neutral; Red: Fear.

**Figure 5:**
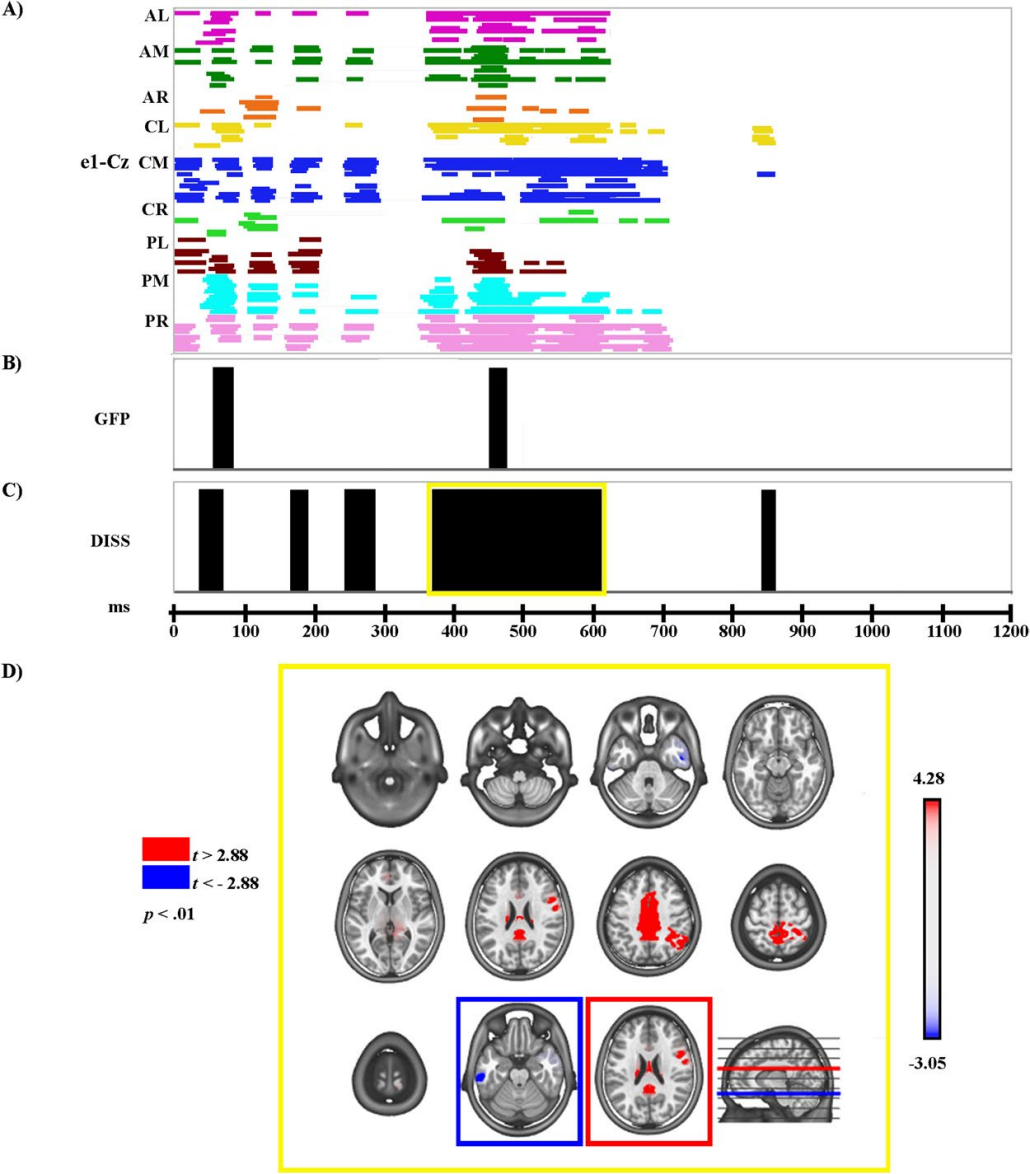
Electrophysiological results and statistical comparison of LAURA source estimation between Happinnes and Neutral over significant TANOVA LPP time interval. (**A**) Statistical analysis of global ERP amplitude. Periods of significant differences of ERP amplitude (*p* < 0.01; duration >20 ms) at each electrode and time-point between conditions are displayed as coloured horizontal lines. Each horizontal line represents one scalp electrode. Different colours indicate different clusters of electrodes (as shown in Fig. 2); AL: anterior left; AM: anterior midline; AR: anterior right. CL: central left; CM: central midline; CR: central right. PL: posterior left; PM: posterior midline; PR: posterior right. (**B**) Global scalp electric field analysis: statistical analysis of global electric field strength. Black areas indicate time intervals of significant differences (*p* < 0.01; duration >20 ms) of Global Field Power (GFP) between conditions. (**C**) Global scalp electric field analysis: statistical analysis of global electric field topography (topographic analysis of variance, TANOVA). Black areas indicate time intervals of significant differences (*p* < 0.01; duration > ms) of global spatial dissimilarity index (DISS) between conditions. (**D**) Significant TANOVA time interval (372–612 ms after Face_2 onset). All significant voxels are coloured (t_(18)_ > 2.88/< −2.88, *p* < 0.01): positive *t* values (red) indicate higher current source densities in Happiness than in Neutral; negative *t* values (blue) indicate higher current source densities in Neutral than in Happiness. LAURA solutions are rendered on MNI152 template brain (left hemisphere on the left side).

**Figure 6:**
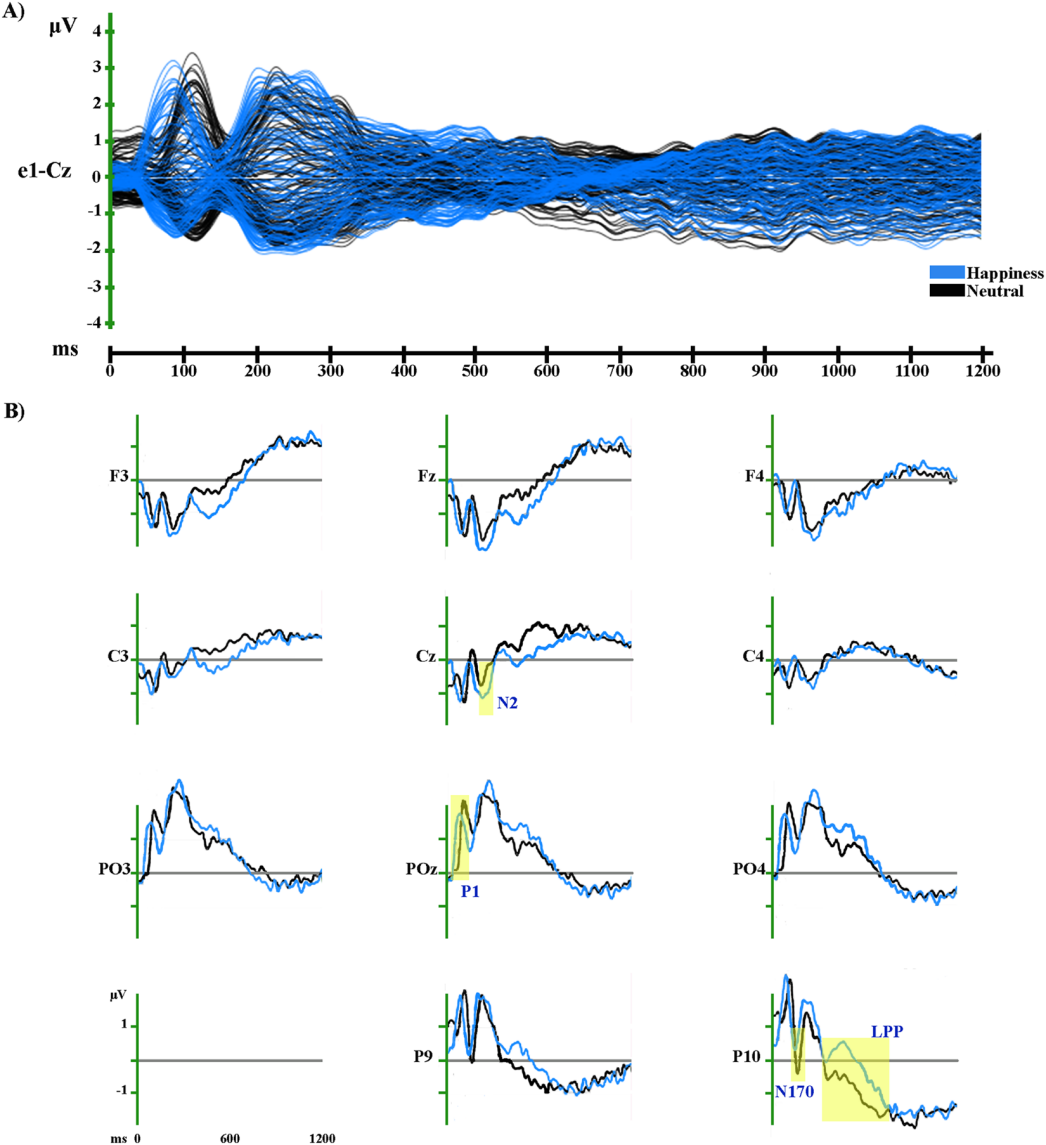
Grand-averaged ERP waveforms of Happy and Neutral conditions. (**A**) Group-averaged (n = 19) event related potential (ERP) waveforms of the two experimental conditions (Happiness and Neutral), superimposed across the 110 recording channels (e1-Cz). (**B**) Group-averaged (n = 19) Face_2-locked ERP waveforms recorded at left, midline and right scalp sites (frontal: F3, Fz, F4; central: C3, Cz, C4; parieto-occipital: PO3, POz, PO4) and at left and right occipito-temporal scalp sites (P9, P10), plotted as voltage in µv and as function of time in ms (Face_2: 0 ms). Black: Neutral; light blue: Happiness.

**Figure 7:**
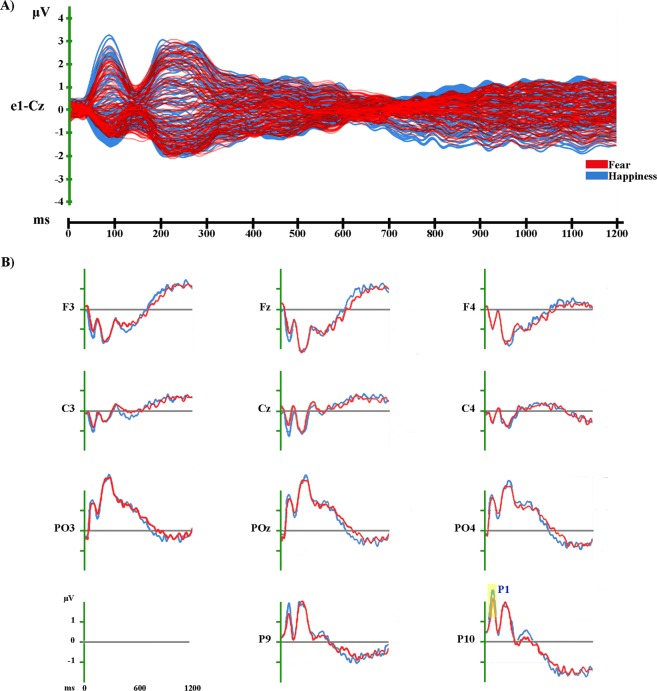
Grand-averaged ERP waveforms of Fear and Happy conditions. (**A**) Group-averaged (n = 19) event related potential (ERP) waveforms of the two experimental conditions (Fear and Happiness), superimposed across the 110 recording channels (e1-Cz). (**B**) Group-averaged (n = 19) Face_2-locked ERP waveforms recorded at left, midline and right scalp sites (frontal: F3, Fz, F4; central: C3, Cz, C4; parieto-occipital: PO3, POz, PO4) and at left and right occipitotemporal scalp sites (P9, P10), plotted as voltage in µv and as function of time in ms (Face_2: 0 ms). Red: Fear; light blue: Happiness.

The electrophysiological results of global ERP waveform analysis, scalp electric field analyses, and source estimations in the LPP time period for Face_2 are reported separately for each comparison (see Figs 3–7).

The electrophysiological results of global ERP waveform analysis for Face_1, performed as an additional control, categorization analyses and results, as well as participant answers about their experience and familiarity with the stimuli, are described in Supplementary Information and Supplementary Fig. S1.

### Fear vs. Neutral

#### Electrophysiological Results

The amplitude analysis showed significant differences in six main time windows (see Fig. 3A). (1) At the onset of Face_2, a significant modulation emerged, likely due to the visual transition from the Object shot to the neutral face that could be explained by the absence of an inter-stimulus interval. (2) From 50 to 80 ms after Face_2 onset a stronger positivity, compatible with a P1 component, was observed in the Fear condition over posterior and central electrodes at a midline location (around POZ; *p* < 0.001; *t* = 5.94). (3) From 100 to 142 ms after Face_2 onset, more positive amplitudes were recorded in the Neutral condition over bilateral posterior electrodes (around O1; *p* < 0.001; *t* = −6.83), and since this significant activity was also compatible with a P1 modulation, the findings in these two time windows could be explained by a difference of latency between the Fear and Neutral conditions during this early stage of processing, with faster processing for Fear than for Neutral (see Fig. 4B). (4) From 168 to 204 ms after Face_2 onset a stronger negativity, compatible with a N170 modulation, was observed in the Neutral condition over bilateral posterior electrodes (around P10; *p* < 0.001; *t* = 6.53) (see Fig. 4B). (5) From 386 to 720 ms after Face_2 onset a stronger positivity, compatible with a LPP, was observed in the Fear condition over posterior electrodes at midline and right locations (around P10; *p* < 0.001; *t* = 5.98) (see Fig. 4B). (6) From 768 to 796 after Face_2 onset a stronger positivity, compatible with a late LPP, was observed in the Fear condition over posterior electrodes at midline and right locations (around P10; *p* < 0.001; *t* = 4.76) (see Fig. 4B). All the significant differences described above, were confirmed by the randomization test.

The GFP analysis (see Fig. 3B) showed three periods of sustained difference between conditions: (1) from 62 to 80 ms; (2) from 108 to 134 ms; and (3) from 380 to 410 ms after Face_2 onset.

The TANOVA (see Fig. 3C) revealed five phases of significant topographic difference between conditions: (1) from 1 to 78 ms; (2) from 102 to 140 ms; (3) from 168 to 192 ms; (4) from 432 to 452 ms; and (5) from 494 to 702 ms after Face_2 onset.

#### Source estimations

During the phase of significant LPP topographic modulation (494–702 ms after Face_2 onset), significantly higher activity in Fear as compared with the Neutral condition (see Fig. 3D, in red; Table 1) was found in various cerebral regions, including a bilateral medial cluster encompassing the premotor cortex (PMC), supplementary motor area (BA6, BA8), middle and posterior cingulate gyri (MCC, PCC; BA24, BA31, BA32), and secondary sensorimotor cortex/superior parietal lobule (BA5, BA7). Furthermore, higher activity in Fear was also found in a right inferior frontal cluster encompassing the IFG (BA9, BA 44), though it comprised only nine contiguous activated solution points and our cut-off was ten solution points. In the Neutral condition, higher activity (see Fig. 3D, in blue; Table 1) was found in left inferior and middle temporal gyri and in the fusiform gyrus (BA 20, 21).

**Table 1:** Source localization of topographic maps.

Condition	TANOVA LPP time period	*t* value	*p* value	Talairach coordinates (x, y, z) mm	Brain region label
Fear > Neutral	494–702 ms	5.23	0.0000	−3, 18, 45	Left medial frontal gyrus, BA 8
		3.78	0.0014	43, 4, 21	Right inferior frontal gyrus, BA 9
Neutral > Fear		−3.69	0.0017	−69, −30, −18	Left middle temporal gyrus, BA 21
Happy > Neutral	372–612 ms	4.28	0.0000	49, 4, 21	Right inferior frontal gyrus, BA 9
		4.16	0.0006	9, −39, 48	Right paracentral lobule, BA 5
		4.05	0.0007	36, −47, 36	Right inferior parietal lobule, BA 40

Significant results of the statistical comparisons of LAURA source estimations in significant TANOVA LPP time periods are reported, with *t* and *p* values, Talairach and Tournoux coordinates (x, y, z) and anatomical labels of solution points with the local maximum different activities. BA = Brodmann Area.

### Happiness vs. Neutral

#### Electrophysiological Results

The amplitude analysis showed significant differences in six main time windows (see Fig. 5A). (1) Like in the previous comparison, at the onset of Face_2 a significant modulation emerged, likely due to the visual change from the Object shot to the neutral face. (2) From 52 to 96 ms after Face_2 onset a stronger positivity, compatible with a P1 component, was observed in the Happiness condition over posterior electrodes at a midline location (around Pz; *p* < 0.001; *t* = 9.33). (3) From 100 to 144 ms after Face_2 onset more positive amplitudes were recorded in the Neutral condition over bilateral posterior electrodes (around O1; *p* < 0.001; *t* = −5.97). Since this significant activity was compatible with a P1 modulation, the findings in these two time windows could be explained by a difference of latency between the Happiness and Neutral conditions during this early stage of processing, with faster processing for the Happiness condition than for Neutral (see Fig. 6B). (4) From 166 to 206 ms after Face_2 onset a stronger negativity, compatible with a N170 modulation, was observed in the Neutral condition over bilateral posterior electrodes (around O1; *p* < 0.001; *t* = 5.01) (see Fig. 6B). (5) From 244 to 286 ms after Face_2 onset a stronger negativity, compatible with a N2 component, was recorded in the Happiness condition over anterior and central electrodes at a midline location (around Cz; *p* < 0.001; *t* = −5.68) (see Fig. 6B). (6) From 354 to 706 ms after Face_2 onset a stronger positivity, compatible with a LPP, was observed in the Happiness condition over posterior electrodes at midline and right locations (around PO8; *p* < 0.001; t = 7.42) (see Fig. 6B). All the significant differences described above, were confirmed by the randomization test.

The GFP analysis (see Fig. 5B) showed two periods of sustained difference between conditions: (1) from 58 to 84 ms and (2) from 454 to 476 ms after Face_2 onset.

The TANOVA (see Fig. 5C) revealed five phases of significant topographic difference between conditions: (1) from 38 to 70 ms; (2) from 168 to 192 ms; (3) from 246 to 288 ms; (4) from 372 to 612 ms; and (5) from 844 to 862 ms after Face_2 onset.

#### Source estimations

For the time period of significant LPP topographic modulation (372–612 ms after Face_2 onset), significantly higher activity in the Happiness as compared with Neutral condition (see Fig. 5D, in red; Table 1) was found in several cerebral regions, including a right inferior frontal cluster which includes the IFG (BA9, BA44), PMC (BA6) insula (BA13), and a bilateral medial cluster encompassing the MCC, PCC (BA23, BA24, BA31, BA32), PMC (BA6) and secondary sensorimotor cortex (BA5), extending to the right inferior and superior parietal lobule (BA7, BA40) and postcentral gyrus (BA3, BA5).

In the same time period, no significantly stronger activations were found in the Neutral condition.

### Fear vs. Happiness

#### Electrophysiological Results

The amplitude analysis showed significant differences in one time window only: from 40 to 126 ms after Face_2 onset, in which a stronger positivity (compatible with a P1 component) was observed in the Happiness condition over posterior electrodes on the right side (around T6; *p* < 0.001; *t* = −5.04) (see Fig. 7). This significant difference was confirmed by the randomization test.

The GFP analysis showed one period of sustained difference between conditions from 810 to 828 ms after Face_2 onset.

The TANOVA revealed one phase of significant topographic difference between conditions from 282 to 300 ms after Face_2 onset.

### Behavioural results

The model explained 29% of the variance in score taking into account the random effects (R^2^_m_ = 0.06; R^2^_c_ = 0.29). More specifically, the variability (*SD*) explained by intercept for subjects and by subject slope for the interaction effect of Context by Measure was 0.29, and <1.04, respectively.

**Figure 8:**
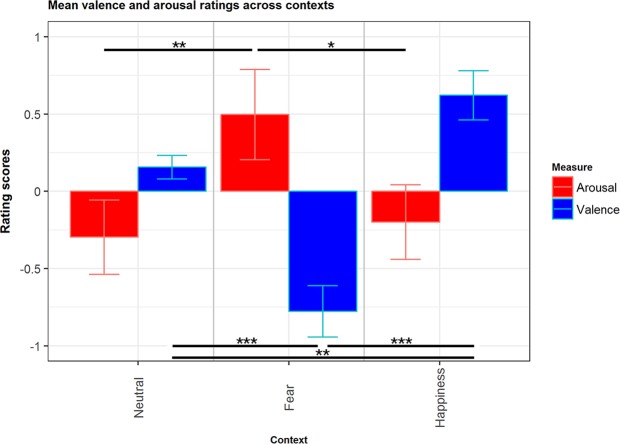
Bar plots of mean valence and arousal ratings across contexts. Error bars represent SE. *p ≤ 0.05; **p ≤ 0.01; ***p ≤ 0.001.

The model revealed a main effect of Context (across both measures) (F_(2,10887)_ = 4.80, *p* = 0.01), with neutral faces in the Fearful context on average being rated 0.79 point higher than neutral faces in the Neutral context (β = 0.79, SE = 0.22, t = 0.6, *p* = 0.0003), while neutral faces in the Happy context did not differ from neutral faces in the neutral context (β = 0.09, SE = 0.1, t = 0.9, *p* = 0.35). The model also revealed a significant Context*Measure interaction effect (F_(2,10887)_ = 10.06, *p* = 0.0001). Post-hoc tests showed that considering Arousal scores, neutral faces in the Fearful context were rated 0.79 point higher than neutral faces in the Neutral context (*p* = 0.005), and 0.69 points higher than neutral faces in the Happy context (*p* = 0.02). There was no significant difference between Neutral and Happiness. Considering Valence scores, neutral faces in the Fearful context were rated −0.93 point lower than neutral faces in the Neutral context (*p* = 0.0001), and −1.4 point lower than neutral faces in the Happy context (*p* < 0.0001). Moreover, neutral faces in the Happy context were rated 0.46 point higher than neutral faces in the Neutral context (*p* < 0.003) (see Fig. 8).

## Discussion

The present study aimed to explore the neural correlates and the time course of the influence of situational contexts on the interpretation of facial expressions. To do this, we used the so-called “Kuleshov effect” in an EEG investigation for the first time.

To this purpose, we used film sequences (created by editing together three different shots) as the stimuli: namely, the close-up of a target person’s neutral face (first Glance shot or Face_1), followed by a view of the scene that the target person was looking at (Object shot; Happiness, Fear, or Neutral), followed by another close-up of the same target person’s neutral face (second Glance shot or Face_2)^37^. We recorded electrophysiological indexes while participants rated both valence and arousal of the target person’s emotion as displayed in Face_2. Subsequently, in a behavioural session, participants were also asked to explicitly provide a categorization, choosing among different labels of emotions.

We analysed the EEG activity evoked by Face_2, comparing each emotional condition with the Neutral condition (Fear vs. Neutral and Happiness vs. Neutral) as well as the two emotional conditions together (Fear vs. Happiness). Results confirmed the presence of a significant modulation of both the N170 and the LPP, the first being of higher amplitude in response to neutral faces preceded by neutral contexts, while the LPP showed higher amplitude in response to neutral faces preceded by emotional contexts (both Happiness and Fear) (see Figs 4 and 6).

Regarding the N170, our results suggest that there is no emotional modulation of this component, as in this case the N170 amplitude should have been higher for neutral faces preceded by emotional contexts. Previous ERP studies investigating the time course of the integration process between facial expressions and other contextual cues reported a clear affective congruence effect on the N170^27,30,31^. For instance, in an affective priming paradigm, Hietanen and Astikainen^27^ investigated the modulation of the N170 by the affective congruence between an emotional facial expression (target: happy or sad) and the affective content of a preceding scene (prime: positive or negative). Results showed that the N170 amplitude was higher when there was an affective congruence between the facial expressions and the emotional scenes (e.g., a positive scene matched with a happy face and a negative scene matched with a sad face). Although we did not employ a priming paradigm (i.e., the “triadic structure” of the stimuli and their duration differed from classic priming paradigms, leading to an explicit evaluation of each shots), taking into account the sequentiality of the three shots, it is possible that there was an affective congruency effect: the N170 amplitude was in fact higher when the neutral face was preceded by a congruent neutral context than when it was preceded by an incongruent emotional (happy or fearful) context (see Figs 4 and 6).

The results of the LPP, being of higher amplitude for neutral faces preceded by emotional contexts, could be interpreted in the same way, since previous ERP literature has widely demonstrated the sensitivity of this component to the *evaluative significance of a stimulus*^36^ (page 795), as well as to the evaluative/affective incongruence with a preceding context^27,33–36^. For instance, Herring *et al*.^36^ employed a sequential evaluative priming paradigm in three different experiments with either affective picture or word pairs. In all cases, the LPP amplitude was higher for evaluative incongruent targets (e.g., a pleasant picture preceded by an unpleasant picture), building on previous results which showed this evaluative effect on the LPP amplitude with both emotional facial and verbal targets^34,35^.

As mentioned previously, Diéguez-Risco and colleagues^33^ presented sentences describing happiness- or anger-inducing situations before the appearance of a congruent or incongruent emotional face. Their results demonstrated the presence of an LPP of higher amplitude for incongruent targets, showing how this component, being related to an affective congruency effect, could be influenced by preceding situational information. The authors discussed this effect as reflecting “*the detection of a discrepancy between the expectation set by the context and the valence of the actual expression shown by the target face*”^33^ (p. 613).

Hence, considering both the sequentiality of the three shots and the rating task of valence and arousal requested of the participants, in our opinion, the present LPP results could be explained in terms of an evaluative congruence effect, and not of an emotional modulation caused by the preceding context on the perception of facial expressions. The fact that the LPP is of higher amplitude for neutral faces preceded by emotional contexts (happy and fearful) in comparison to neutral faces preceded by neutral contexts suggests the occurrence of a violation of expectations established by the context itself.

In our opinion, this explanation is further supported by the absence of significant results in the LPP time window when comparing the two emotional conditions (i.e. Happiness vs. Fear) (see Fig. 7), thus excluding the possibility that it could be explained by an emotional or attentional effect^18–21^.

In this case the LPP amplitude should have been higher for neutral faces preceded by contexts of high emotional arousal (i.e., Fear condition) than for neutral faces preceded by contexts of lower emotional arousal (i.e. Happiness condition).

The global scalp electric field analysis revealed that for both comparisons (Fear vs. Neutral and Happiness vs. Neutral) the LPP modulation was characterized by topographic differences between the two conditions (see Figs 3C and 5C). Significantly higher activity in the Fear and Happiness conditions was found in a medial cluster encompassing the cingulate cortex (MCC, PCC) and the PMC (Figs 3D and 5D). Regarding the MCC and PCC, previous studies demonstrated their involvement in the processing of facial expressions and scenes during emotional tasks, as well as in the interaction between emotion and episodic memory^6,61–63^. On the other hand, the activation of the PMC could suggest the involvement of resonance mechanisms promoting the processing of motor information conveyed by images of facial expressions^64,65^.

Concerning this latter result, higher activity in the emotional conditions was also found in the right IFG (Figs 3D and 5D), which is involved not only in the processing of facial expressions^6,10^ but also in the comprehension of others’ motor intentions through a visual-motor resonance mechanism^64,65^. It is worth noting that only in the Happiness vs. Neutral comparison was higher activity in the Happiness condition also found in the inferior parietal regions (i.e. the inferior parietal lobule (IPL) and intraparietal sulcus) and postcentral gyrus/primary somatosensory cortex (BA3).

In contrast, in the Fear vs. Neutral comparison only, the Neutral condition revealed a significantly higher activation of the left inferior and middle temporal regions.

Bearing in mind that these data refer to the LPP time window of Face_2 (during which participants explicitly evaluated its valence and arousal), it seems that the processing of neutral faces preceded by emotional contexts (incongruent conditions) requires the involvement of regions underpinning the processing of facial expressions and emotion recognition. Furthermore, PMC and right IFG activation could signal the involvement of motor resonance mechanisms which are thought to facilitate intersubjective empathy. The additional involvement of the IPL and postcentral gyrus/primary somatosensory cortex in the Happiness condition could be explained by the behavioural results (see below), which suggest that Happy contexts did not differ from the Neutral ones in terms of arousal or salience. Thus, if the incongruence between Happy contexts and neutral faces is less explicit, the additional recruitment of these regions may be necessary for effective recognition of happy emotional facial expressions.

Beyond the N170 and the LPP, a significant modulation was also found in the early time window of the P1 component (a marker of low-level feature processing)^28^. In our opinion, this modulation is better explained as a latency effect with faster processing of neutral faces preceded by emotional contexts (both Fear and Happiness), likely due to a higher attentional engagement set by the emotional content of the contexts. However, since between the context and the neutral facial expression there was no inter-stimulus interval, we should also consider the possibility that in this case the P1 does not reliably reflect the early low-level processing of faces.

In addition, in the Happiness vs. Neutral comparison we also found a significant modulation of the anterior N2 component, with a higher amplitude for neutral faces preceded by Happy contexts than for neutral faces preceded by Neutral contexts (see Fig. 6). It is noteworthy that, although there was no significant difference between the two conditions, the same modulation was also visible for the Fear vs. Neutral comparison (see Fig. 4). Previous studies have shown that this is related to conflict monitoring in face processing^66,67^_._ Namely, when investigating the processing of emotional conflict between faces and scenes, Xu *et al*.^67^ found that the amplitude of the fronto-central N2 evoked by incongruent face-scene compound stimuli was higher than the one evoked by congruent stimuli. Hence, our results could reflect a similar case of emotional conflict monitoring between the context and the subsequent facial expression.

Regarding the behavioural results, there was a significant effect of Fear and Happy contexts on valence, and of Fear context only on arousal scores: participants rated neutral faces preceded by Fearful contexts as significantly more negative than neutral faces preceded by both Neutral or Happy contexts, and neutral faces preceded by Happy contexts as significantly more positive then neutral faces preceded by Neutral contexts. Furthermore, participants rated neutral faces preceded by Fearful contexts as more arousing than neutral faces preceded by both Neutral or Happy contexts. Finally, participants categorized the target person’s emotion congruently with the preceding context (see Supplementary Information).

In light of the aforementioned EEG and behavioural results, the present findings suggest that with this specific paradigm, perceptual experience of a neutral facial expression may not be clearly disambiguated by the context. Thus, there is no “projection” of the contextual emotional content on Face_2, but rather a more cognitive process related to expectations. Indeed, the N170 and the LPP exhibited sensitivity to the affective and evaluative congruence between the context and the subsequent neutral facial expression, yet there was no difference between the two emotional conditions.

These results suggest that the “Kuleshov effect” could be explained by a cognitive process of attribution of expectations set by the context itself and not by an actual perceptual and emotional experience. In our previous behavioural study^37^, we hypothesized that, *“The context triggers the arousal and the emotional reaction in the observer*, *who then attributes an emotional value to a neutral face”*. In light of the present findings, we can continue to maintain our claim that the context sets expectations of how one would feel in a particular situation (in terms of the emotional reaction displayed through the face). At the brain level, these expectations are violated by the subsequent neutral facial expression, but on the behavioural level the observer, on the basis of the same expectations, attributes an emotional value to the same neutral facial expression. However, taking into account the novelty of the present paradigm, more evidence is needed to confirm our claim. First of all, the role of Face_1 has to be clarified. Future investigations are needed to disentangle how Face_1 may interact with the context (e.g. Face_1 could prime neutral contexts more than emotional contexts, causing subsequent knock-on effects on EEG responses to Face_2). Furthermore, to shed light on whether Face_1 could have enhanced the influence of the context in its implication that the actor is looking at the Object shot (see Introduction section), future studies could compare this Kuleshov paradigm with one in which Face_1 is omitted.

Secondly, in order to comprehensively test for the affective and evaluative congruence-incongruence effect, although it would represent a deviation from the original Kuleshov experiment, a good option for future studies would be to manipulate the expression of Face_2 by adding happy and fearful conditions.

Furthermore, taking into account that between the context and the neutral facial expression there was no inter-stimulus interval, we cannot rule out that differences among the Object shots (e.g. differences in luminance, in content and so on) may have carry-over effects on early ERP components. Lastly, we cannot rule out the possibility that the participants’ explicit judgments were influenced by social desirability factors or by their assumption of the experimenter’s expectations with regard to their answers^38^. To avoid this possible source of error and to gain insight into between-participant variability of how the semantic gap between face and context can be filled, future studies could adopt a more nuanced approach to collecting behavioural data. For example, whereas a categorization task may lead to induced or limited responses and demand characteristics, open-ended questions would invite the participants to express his or her own particular phenomenological experience. An even more ecological approach with regard to intersubjective experience, as well as to the aesthetic parameters of the Kuleshov effect, could be pursued by using emotionally ambiguous faces instead of expressionless or neutral ones.

## Supplementary information

Supplementary Information File

## Data Availability

The data that support the findings of this study are available from the corresponding author upon reasonable request.
