# Supplementary material for: How context influences the interpretation of facial expressions: a source localization high-density EEG study on the “Kuleshov effect”
Source: Sci Rep. 2019 Feb 14;9:2107. doi: 10.1038/s41598-018-37786-y (PMC6376122; doi:10.1038/s41598-018-37786-y)
Supplement: Supplementary file 1 — Supplementary Information File [file 41598_2018_37786_MOESM1_ESM.pdf]

How context influences the interpretation of facial expressions: a source localization high-density EEG study on the "Kuleshov effect".

Marta Calbi[1*], Francesca Siri[1], Katrin Heimann[2], Daniel Barratt[3], Vittorio Gallese[1,4*], Anna Kolesnikov[5], Maria Alessandra Umiltà[6]

[1] Department of Medicine and Surgery, Unit of Neuroscience, University of Parma, Parma, Italy

[2] Interacting Minds Center, University of Aarhus, Aarhus, Denmark

[3] Department of Management, Society and Communication, Copenhagen Business School, Copenhagen, Denmark

[4] Institute of Philosophy, School of Advanced Study, University of London, London, UK

[5]Department of Humanities, Social Sciences and Cultural Industries, University of Parma, Parma, Italy.

[6] Department of Food and Drug Sciences, University of Parma, Parma, Italy

* Corresponding author

E-mail: calbimarta@gmail.com

vittorio.gallese@unipr.it

Assessment

In order to exclude the possibility that personality traits or deficits in emotion recognition and empathic abilities could influence task performance, one day before the experimental session, participants were asked to fill in several questionnaires via Google Forms: the Toronto Alexithymia Scale (TAS-20), which measures the ability to identify and describe emotions and feelings, and has three subscales (Difficulty Describing Feelings (DDF), Difficulty Identifying Feelings (DIF) and Externally-Oriented Thinking (EOT)[68]; the Interpersonal Reactivity Index (IRI), which assesses the empathic abilities of each participant, and has four subscales (Perspective Taking (PT), Fantasy (FS), Empathic Concern (EC) and Personal Distress (PD))[69]; and the Behavioural Activation System/Behavioural Inhibition System (BIS/BAS), which measures individual differences in the sensitivity of these systems, and has one BIS-related scale and three BAS-related scales (BAS Drive, BAS Fun Seeking and BAS Reward Responsiveness)[70]. Once participants arrived at the laboratory, they were also asked to fill in the State-Trait Anxiety Inventory (STAI)[71], in order to assess

the level of anxiety of each participant as a permanent trait and/or as contextual. Below are the mean values obtained in each questionnaire by the 19 participants whose EEG data were analysed:

TAS-20: the mean DDF subscale score ± SD was 11.37 ± 4.80; the mean DIF subscale score ± SD was 13.80 ± 7.4; the mean EOT subscale score ± SD was 13.11 ± 4.20; the mean total score ± SD was 38.30 ± 13.45;

IRI: the mean EC score ± SD was 19.90 ± 5.05; the mean PD score ± SD was 10 ± 4.53; the mean PT score ± SD was 17.80 ± 4.90; the mean FS score ± SD was 16.74 ± 5.45.

BIS/BAS: the mean BIS score ± SD was 24.11 ± 3.80; the mean BAS Drive score ± SD was 13.60 ± 2.70; the mean BAS Fun Seeking score ± SD was 12.63 ± 3.17; the mean BAS Reward Responsiveness score ± SD was 21.11 ± 3.05;

STAI: the mean STAI X2 Trait score ± SD was 42.32 ± 7.51; the mean STAI X1 Pre score ± SD was 34.30 ± 6.40; the mean STAI X1 Post score ± SD was 16 ± 3.50;

Categorization task analysis and results

If the emotional contexts had no effect on the emotional attribution of the target person's emotional state (the null hypothesis), then each of the seven categories should have been selected with an equal degree of probability[37,38]; that is, a relative frequency approaching 14.3%.

For the Fearful context, participants tended to choose negative emotions more frequently than the other options (Sadness=24.2%, Fear=23.5%, Disgust=15%, Anger=12.8%, Surprise=11.3%, Happiness=9.5%, Other=3.6%).

For the Happy context, participants tended to choose happiness more frequently than the other options (Happiness=42.6%, Sadness=17.3%, Surprise=13.4%, Other=8.1%, Anger=7%, Fear=6%, Disgust=5.7%).

For the Neutral context, participants tended to choose both happiness and sadness more frequently than the other options (Happiness=25.2%, Sadness=23.7%, Other=14.1%, Surprise=12.8%, Anger=11.1%, Fear=8.3%, Disgust=5%).

To further examine the relation among participants' answers across the three different contexts, a Pearson's chi-square test of independence was performed. As expected, the associations among these variables were significant ($\text{Chi-Square}_{(12,\ 5184)} = 911.5979$, $p <.01$). Furthermore, by inspecting the individual cells in Crosstabulation in Supplementary Table S1, it emerges that, for the Fearful context, participants tended to choose negative emotions more frequently than the other options (Sadness=24.2%, Fear=23.5%, Disgust=15%, Anger=12.8%, Surprise=11.3%, Happiness=9.5%, Other=3.6%). Specifically, the cells associated whit negative emotions had positive adjusted standardized residual values (Sadness=3.1, $p < .01$; Fear=16.8, $p < .001$; Disgust=11.7, $p < .001$; Anger=4.3, $p < .001$), distributed according to the Standard Normal Distribution (Mean = 0, Standard Deviation = 1, cutoff point set at |3| with $p < .01$), indicating that participants chose negative emotions significantly more frequently than what would be expected by chance.

In the opposite way, the cell associated with Happiness had negative adjusted standardized residual values (Happiness = -18.9, $p < .001$), indicating that participants chose Happiness significantly less frequently than what would be expected by chance.

For the Happy context, participants tended to choose Happiness more frequently than the other options (Happiness=42.6%, Sadness=17.3%, Surprise=13.4%, Other=8.1%, Anger=7%, Fear=6%, Disgust=5.7%). The cell associated with Happiness had, indeed, positive adjusted standardized residual values (Happiness=19.6, $p < .001$), indicating that participants chose Happiness significantly more frequently than what would be expected by chance. On the other hand, the cells associated whit negative emotions had negative adjusted standardized residual values (Sadness = -5.5, $p < .001$, Fear = -10.2, $p < .001$, Disgust = -5.2, $p < .001$, Anger = -5.6, $p < .001$), indicating that participants chose negative emotions less frequently than what would be expected by chance.

For the Neutral context, participants tended to choose both Happiness and Sadness more frequently than the other options (Happiness=25.2%, Sadness=23.7%, Other=14.1%, Surprise=12.8%, Anger=11.1%, Fear=8.3%, Disgust=5%). It is worth noting that, in this context the cells associated whit Disgust and Fear had negative adjusted standardized residual values (Disgust = -6.5, $p < .001$, Anger = -6.6, $p < .001$), indicating that participants chose these two emotions less frequently than what would be expected by chance.

For all analyses, we used R (R Core Team, 2012) and descr[72].

**FEAR**	**Anger**	**Disgust**	**Fear**	**Happiness**	**Sadness**	**Surprise**	**Other**
Observed	222	258	406	165	419	195	63
Expected	178.00	147.00	217.00	445.67	375.67	215.67	149.00
ChiSq.	10.88	83.82	164.61	176.75	5.00	1.98	49.64
Adj. std. res.	4.26	11.72	16.80	-18.90	3.10	-1.84	-9.03
**HAPPINESS**							
Observed	120	98	102	737	299	231	141
Expected	178.00	147.00	217.00	445.67	375.67	215.67	149.00
ChiSq.	18.90	16.33	60.94	190.45	15.65	1.09	0.43
Adj. std. res.	-5.62	-5.17	-10.22	19.62	-5.48	1.37	-0.84
**NEUTRAL**							
Observed	192	85	143	435	409	221	243
Expected	178.00	147.00	217.00	445.67	375.67	215.67	149.00
ChiSq.	1.10	26.15	25.24	0.26	2.96	0.13	59.30
Adj. std. res.	1.36	-6.55	-6.58	-0.72	2.38	0.48	9.87

Supplementary Table S1

Electrophysiological results

Global ERP waveform analysis Face_1

Results of the global ERP waveform analysis for Face_1, performed as an additional control, were not significant for any of the considered comparisons (Fear vs. Neutral, Happiness vs. Neutral, Fear vs. Happiness), thus confirming that significant modulations found for Face_2 could be explained only by the emotional scenes' content (Object-shot). For a comparison between global ERP waveform analysis for Face_1 and for Face_2, please see Supplementary Figure S1.

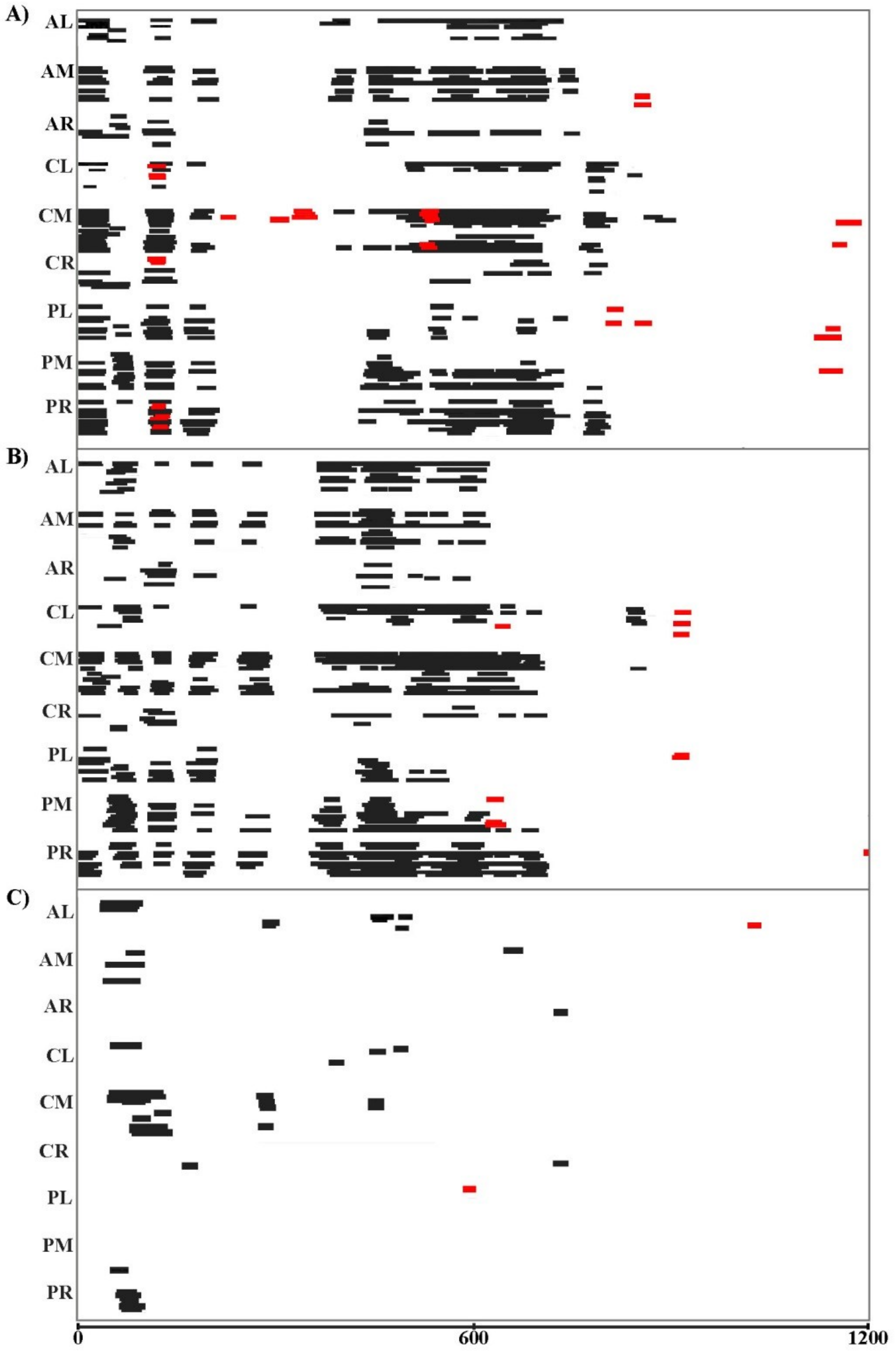

Supplementary Fig.S1

Superimposed statistical analyses of global ERP amplitude on Face_1 (in red) and Face_2 (in black) data for each comparison. Periods of significant differences of ERP amplitude ($p < .01$; duration > 20 ms) at each electrode and time point between conditions are displayed as horizontal lines. Each horizontal line represents one scalp electrode. AL: anterior left; AM: anterior midline; AR: anterior right. CL: central left; CM: central

midline; CR: central right. PL: posterior left; PM: posterior midline; PR: posterior right. A) Fear vs. Neutral; B) Happy vs. Neutral; C) Fear vs. Happy.

ERP analyses and results – N170 and LPP mean amplitude

The mean amplitude of the posterior N170 response was measured at occipito-temporal (P9, P10) and occipital (O1, O2) sites between 160 and 210 ms after Face_2 onset. The mean amplitude of the posterior LPP was measured at occipito-temporal sites (PO7, P9, PO8, P10) in the 350-720 time window. ERP data were subjected to multifactorial repeated-measures ANOVAs with two within group factors: Condition (Neutral, Happiness, Fear) and Hemisphere (Left, Right). Multiple comparisons of the means were performed with Tukey's post-hoc tests. All data showed normal distribution in accord with Shapiro-Wilk test. The Mauchley test did not reveal any violation of sphericity.

N170: The ANOVA revealed a significant main effect of Condition ($F_{(2,36)}$=26,683; $p < .001$). Tukey post-hoc tests showed that the mean amplitude elicited by Neutral condition was significantly larger than that elicited by both Happiness and Fear conditions (Neutral: Mean - M = 0,89 µV; Standard Error - SE = 0.18. Happiness: M = 1.53; SE = 0.2. Fear: M = 1.70; SE = 0.19) (*Ps* < .00014), while there was no a significant difference between Fear and Happiness conditions ($p = .30$).

LPP: The ANOVA revealed a significant main effect of Condition ($F_{(2,36)}$=19,005; $p < .001$). Tukey post-hoc tests showed that the mean amplitude elicited by Neutral condition was significantly lower than that elicited by both Happiness and Fear conditions (Neutral: M = -0.53 µV; SE = 0.15. Happiness: M = 0.16; SE = 0.2. Fear: M = 0.03; SE = 0.11) (*Ps* < .00022), while there was no a significant difference between Fear and Happiness conditions ($p = .55$). The ANOVA revealed also a significant Condition* Hemisphere interaction ($F_{(2,36)}$=5,2588; $p = .01$). Tukey post-hoc tests showed that the difference between Neutral and Happiness/Fear was significant on both Left and Right hemispheres (*Ps* < .0023). Furthermore, there a was significant difference between Left and Right hemisphere only for Neutral condition ($p = .0035$). (Left = Neutral: M = -0.32 µV; SE = 0.13. Happiness: M = 0.13; SE = 0.2. Fear: M = 0.14; SE = 0.13; Right = Neutral: M = -0.75 µV; SE = 0.2. Happiness: M = 0.18; SE = 0.19. Fear: M = -0.08; SE = 0.15).

ERP analyses and results – N170 peak latency

To investigate the presence of significant differences in the latency of the N170 peak among conditions, we performed a repeated-measures ANOVA on the latency of the N170 peak, with two within group factors: Condition (Neutral, Happiness, Fear) and Hemisphere (Left, Right). The latency was measured at occipito-temporal (P9, P10) and occipital (O1, O2) sites between 160 and 210 ms after Face_2 onset. Multiple

comparisons of the means were performed with Tukey's post-hoc tests. All data showed normal distribution in accord with Shapiro-Wilk test. The Mauchley test did not reveal any violation of sphericity.
The ANOVA revealed a significant main effect of Condition ($F_{(2,36)}$=35,077; p < .001). Tukey post-hoc tests showed that the N170 occurred significantly later for Neutral condition than for both Happiness and Fear conditions (Neutral: M = 163 ms; SE = 2.5. Happiness: M = 146 ms; SE = 2.9. Fear: M = 142 ms; SE = 3.2) (Ps < .00013). There was no a significant difference between Fear and Happiness conditions (*p* = .33).

Experience and familiarity assessment

At the end of the whole experimental procedure, participants were asked to answer six open questions via Google Forms to assess their previous experience and their familiarity with the stimuli: 1) Have you ever seen these videos before? 2) What do you think the experiment was about? 3) Was there anything confusing in the experiment? 4) What was your impression of the different faces? 5) Do you have any other comments? 6) Have you heard of the Soviet filmmaker Lev Kuleshov and/or the ‘‘Kuleshov effect’’? The table below (Supplementary Table S1) shows the answers from the entire sample of 24 participants.

**Questions**	**Descriptions of answers**
Have you ever seen these videos before?	Only one participant had the impression of seeing some of the emotional contextual scenes before.
What do you think the experiment was about?	Most participants thought it was about *"emotions"*, *"empathy"*, *"facial expressions"*. Some of them also considered the potential *role of context in influencing their perceptions and interpretations of facial expressions*.
Was there anything confusing in the experiment?	While most participants did not find anything confusing, two of them reported that *"some facial expressions did not clearly show an emotion"* or *"the emotional expression was too aseptic, difficult to place in a context"*
What was your impression of the different faces?	Several participants noted that the target faces seemed to be "*similar"* among each other in terms of emotional expressions, "*fairly inexpressive"*, "*relatively sad"*, or "*calm"*.
Do you have any other comments?	Participants did not report relevant comments.
Have you heard of the Soviet filmmaker Lev Kuleshov and/or the ‘‘Kuleshov effect’’	None of the participants knew the "Kuleshov effect"

Supplementary Table S2

References

68. Bagby, R. M., Parker, J. D. A. & Taylor, G. J. The twenty-item Toronto Alexithymia scale—I. Item selection and cross-validation of the factor structure. *J. Psychosom. Res.* **38,** 23–32 (1994).

69. Davis, M. H. A Multidimensional Approach to Individual Differences in Empathy. *J. Pers. Soc. Psychol.* **44,** 113–126 (1983).

70. Carver, C. S. & White, T. L. Behavioral inhibition, behavioral activation, and affective responses to impending reward and punishment: The BIS/BAS Scales. *J. Pers. Soc. Psychol.* **67,** 319–333 (1994).

71. Spielberger, C. D., Gorsuch, R. L., and Lushene, R. E. *Manual for the State-Trait Anxiety Inventory*. (Palo Alto, CA: Consulting Psychologists Press, 1970).

72. Aquino, J. Includes R source code and/or documentation written by Dirk Enzmann, Marc Schwartz, Nitin Jain and Stefan Kraft. descr: Descriptive Statistics. R package version 1.1.4. https://CRAN.R-project.org/package=descr (2018).
